# H3.3-H4 Tetramer Splitting Events Feature Cell-Type Specific Enhancers

**DOI:** 10.1371/journal.pgen.1003558

**Published:** 2013-06-06

**Authors:** Chang Huang, Zhuqiang Zhang, Mo Xu, Yingfeng Li, Zhen Li, Yanting Ma, Tao Cai, Bing Zhu

**Affiliations:** 1 College of Biological Sciences, China Agricultural University, Beijing, China; 2 National Institute of Biological Sciences, Beijing, China; Ludwig Institute for Cancer Research, University of California San Diego, United States of America

## Abstract

Previously, we reported that little canonical (H3.1–H4)_2_ tetramers split to form “hybrid” tetramers consisted of old and new H3.1–H4 dimers, but approximately 10% of (H3.3–H4)_2_ tetramers split during each cell cycle. In this report, we mapped the H3.3 nucleosome occupancy, the H3.3 nucleosome turnover rate and H3.3 nucleosome splitting events at the genome-wide level. Interestingly, H3.3 nucleosome turnover rate at the transcription starting sites (TSS) of genes with different expression levels display a bimodal distribution rather than a linear correlation towards the transcriptional activity, suggesting genes are either active with high H3.3 nucleosome turnover or inactive with low H3.3 nucleosome turnover. H3.3 nucleosome splitting events are enriched at active genes, which are in fact better markers for active transcription than H3.3 nucleosome occupancy itself. Although both H3.3 nucleosome turnover and splitting events are enriched at active genes, these events only display a moderate positive correlation, suggesting H3.3 nucleosome splitting events are not the mere consequence of H3.3 nucleosome turnover. Surprisingly, H3.3 nucleosomes with high splitting index are remarkably enriched at enhancers in a cell-type specific manner. We propose that the H3.3 nucleosomes at enhancers may be split by an active mechanism to regulate cell-type specific transcription.

## Author Summary

In our previous study, we unexpectedly discovered that nucleosomes containing the variant H3.3 histones experience substantial splitting events, resulting hybrid nucleosomes containing both “old” and “new” H3.3–H4 dimers. Here, we mapped the genomic distribution of these splitting events at the genome-wide level and studied the connections among gene transcriptional activity, H3.3 nucleosome occupancy, H3.3 nucleosome turnover and H3.3 nucleosome splitting events. We found that H3.3 nucleosome splitting events are better markers that reflect the transcriptional activity. Moreover, we discovered that H3.3 nucleosome splitting events feature the cell-type specific enhancers, which do not appear to the mere consequence of H3.3 nucleosome turnover. These findings may suggest an active mechanism regulating the H3.3 nucleosome splitting events at the enhancers.

## Introduction

H3.3 is a variant histone that differs from the canonical H3 histones by four amino acids [1]–[4]. Unlike the canonical histones that are incorporated in the replication-dependent pathway, H3.3 histones can also be deposited in a replication-independent manner [5]. Genome-wide profiling experiments in Drosophila cells demonstrated a general enrichment of H3.3 histones at actively transcribing genes [6] and a localized enrichment at the Polycomb responsive elements (PRE) [7]. In mammals, the HIRA complex mediates the incorporation of H3.3 histones at active genes [8], [9] whereas the ATRX-DAXX complex mediates the deposition of H3.3 histones at telomeric and pericentric heterochromatin [9]–[11].

Histone modifications carry important epigenetic information [12]–[14]. Understanding how the patterns of histone modification are transmitted to daughter cells during mitotic division is a highly interesting topic [15]–[20]. We reported that the lysine methylation of histones does not necessarily proceed in a symmetrical fashion within each nucleosome [21] and that canonical (H3.1–H4)_2_ tetramers undergo conservative segregation during replication-dependent chromatin assembly [22]. These studies ruled out a model in which the faithful copying of modifications within each nucleosome serves as the general mechanism for the inheritance of histone modification-based epigenetic information [23]. However, the existence of such a mechanism at specific genomic regions remains possible [24], for example, at certain regulatory sites [25].

Unlike the canonical (H3.1–H4)_2_ tetramers that rarely split, we reported that the (H3.3–H4)_2_ tetramers experience splitting events at a ratio of approximately 10% in each cell division in HeLa cells [22]. Here, we report the mapping of occupancy, turnover rate and splitting events for H3.3 nucleosomes at the genome-wide level. We found a remarkable enrichment of the (H3.3–H4)_2_ tetramer splitting events at cell-type specific enhancers, which may suggest a potential connection between the H3.3 nucleosome splitting and the maintenance of the lineage-specific transcription status.

## Results

### Purification of “hybrid” mononucleosomes that contain newly synthesized and old H3.3–H4 dimers

**Figure 1:**
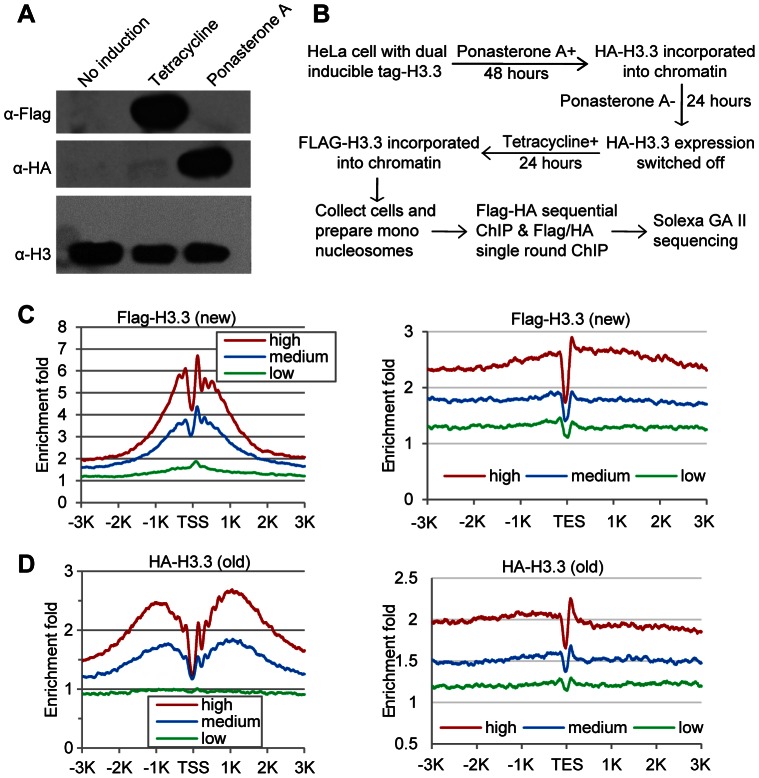
Determine H3.3 nucleosome occupancy, turnover and splitting events at the genome-wide level. (A) Induction of Flag-H3.3 and HA-H3.3 histones. (B) Experimental scheme to determine the splitting index. (C) Distribution profiles of new H3.3 nucleosomes (Flag-tagged) around the TSS (left panel) and TES (right panel). Genes were divided into 3 groups according to their RPKM: High, the top one-third genes; Medium, the middle one-third genes and Low, the bottom one-third genes. (D) Distribution profiles of old H3.3 nucleosomes (HA-tagged).

To map the genome-wide distribution pattern of the (H3.3–H4)_2_ tetramer splitting events, “hybrid” mononucleosomes that contain both newly synthesized and existing old H3.3–H4 dimers must be purified. Accordingly, we established a stable HeLa cell line that contains dual, inducibly expressed H3.3: a Flag-tagged histone H3.3 under the control of a tetracycline-inducible promoter and an HA-tagged H3.3 under the control of a Ponasterone A-inducible promoter (Figure S1). We previously reported that ectopically expressed H3 histones accounted for less than 3% of the total H3 histones [22]. Consistently, the HA- and Flag-tagged H3.3 histones were readily detected by antibodies against these epitope tags; in contrast, the larger version of the ectopically expressed H3.3 histones were barely detectable using antibodies against H3 (Figure 1A).

The Flag-H3.3 and HA-H3.3 histones were then allowed to express at distinct time periods, which designates the HA-H3.3 histones as the “old” H3.3 and the Flag-H3.3 histones as the “new” H3.3 (Figure 1B). Mononucleosomes were prepared from these cells (Figure S2A) and then subjected to chromatin immunoprecipitation (ChIP). The Flag-H3.3 or HA-H3.3 containing mononucleosomes were purified with a single-round ChIP to generate the pools of “new” or “old” H3.3 nucleosomes, respectively, and the split H3.3 nucleosomes were selectively purified by sequential ChIPs with antibodies against the Flag and HA tags (Figure S2B). To ensure that we indeed sequence DNA samples from the split mononucleosomes, the library DNA fragments between 200 and 300 bp were size-fractionated (Figure S2C) prior to single-end sequencing (Figure S2D), because 92 bp of adapter sequences were ligated to the DNA samples during library construction.

### Identification of the H3.3 nucleosomes and scoring the H3.3 nucleosome turnover index

To genome-widely map total H3.3 nucleosomes distribution, sequencing results from the two single-round ChIPs, which consisted of both the old (HA-H3.3) and new (Flag-H3.3) nucleosomes were pooled and analyzed using the sliding-windows method (See the Materials and Methods). In total, we identified 732,944 well-positioned H3.3 nucleosomes.

The genome-wide turnover kinetics for nucleosomes has been reported previously [26]. However, the genome-wide H3.3 nucleosome turnover pattern has not been specifically determined. Because we selectively purified the old (HA-tagged) and new (Flag-tagged) H3.3 nucleosomes (Figure 1B), we were able to compare their genomic profiles. Generally, both the new (Flag-tagged) and old (HA-tagged) H3.3 histones were enriched around the transcription start sites (TSS) and depleted at the transcription end sites (TES) (Figure 1C–1D). However, the old H3.3 nucleosomes displayed a broader cleft around the TSS than the new H3.3 nucleosomes (Figure 1C–1D), suggesting that the H3.3 nucleosomes at the TSS experience a higher turnover rate than the H3.3 nucleosomes located elsewhere.

**Figure 2:**
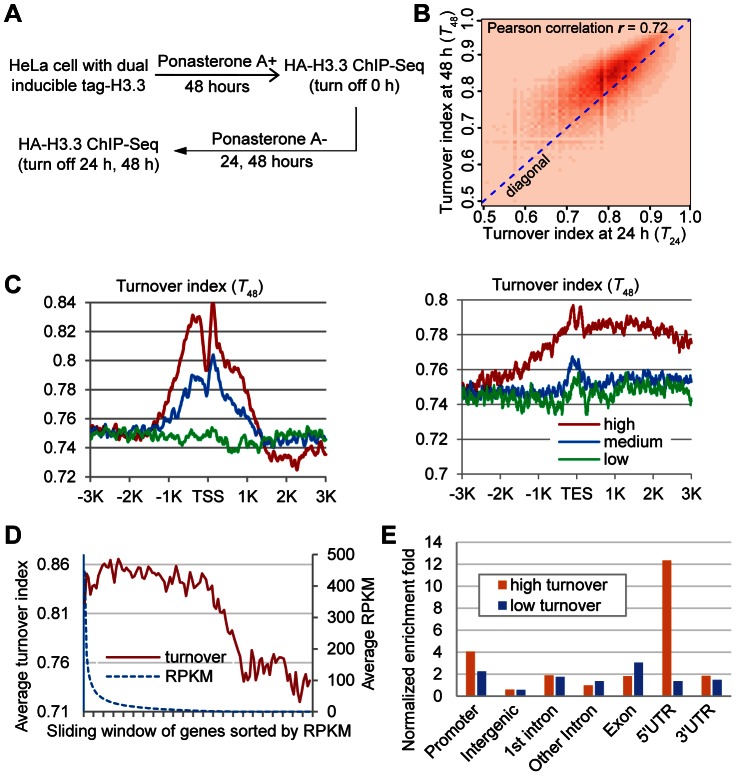
Genome-wide analysis of H3.3 nucleosome turnover. (A) Experimental scheme to determine the turnover index. (B) Two-dimensional histogram of *T_24_* and *T_48_* for all H3.3 nucleosomes. (C) Distribution profiles of the H3.3 nucleosome turnover index around the TSS (left panel) and TES (right panel). (D) Bimodal distribution of turnover at +1 nucleosome versus expression level. Genes were sorted by RPKM from high to low with a sliding widow of 600 genes and then plotted against their turnover index at the +1 nucleosome. (E) Genomic distribution of high turnover and low turnover H3.3 nucleosomes.

The above experiments provided some hints about the turnover of H3.3 nucleosomes. However, these experiments were not specifically designed for determining the turnover rate of H3.3 nucleosomes. We attempted to develop a mathematic model using the above data set, but too many approximations had to be incorporated into the equations, which may affect the accuracy of the model. In order to directly measure the turnover rate of H3.3 nucleosomes, we performed a second set of experiments, in which we induced the expression of HA-H3.3 histones for 48 h and then switched it off (Figure 2A). Cells were harvested at 0 h, 24 h and 48 h after the termination of induction. Mononucleosomes were prepared from these cells and then subjected to ChIP-Seq with antibodies against HA (Figure S2D).

The ChIP-Seq profiles at 24 h and 48 h time points were compared to the ChIP-Seq profile at 0 h to generate the turnover index (*T_24_* and *T_48_*) respectively, for each H3.3 nucleosome (See the Materials and Methods for details). We calculated the Pearson correlation of T_24_ and T_48_ and they displayed clear positive correlation (*r* = 0.72). To directly visualize the above results, we plotted the two-dimensional histogram for *T_24_* and *T_48_* of all H3.3 nucleosomes. Indeed, turnover index at these two time points displayed clear positive correlation, and *T_48_* were generally higher than *T_24_* (Figure 2B). These results collectively reflected the linearity and continuity of H3.3 nucleosome turnover during the tested time window. In all further analysis, we focused on *T_48_* because that was the same time window we used to determine the splitting events and *T_48_* would allow us to perform a direct comparison.

We analyzed the H3.3 nucleosome turnover event along protein coding genes. Indeed, the H3.3 nucleosomes at the TSS regions had higher turnover index and active genes generally displayed higher H3.3 nucleosome turnover (Figure 2C), confirming what we observed earlier (Figure 1C–1D).

### Bimodal distribution of the H3.3 nucleosome turnover at TSS regions

We sorted all of the genes by their expression levels, from high to low, as represented by the RPKM (reads per kb of exon per million mapped reads) obtained from the RNA-Seq experiments, and then plotted the turnover index of the H3.3 nucleosomes at the +1 nucleosome of each gene against its RPKM. The H3.3 nucleosome turnover index at this region displayed a moderate decline for active genes within top 60% expression levels, although the expression levels of these genes could differ for more than 300 fold determined by their RPKM (Figure 2D). H3.3 nucleosome turnover at the TSS regions appeared in a bimodal distribution (Figure 2D) suggesting that genes are either active with high H3.3 nucleosome turnover or silenced with low H3.3 nucleosome turnover, rather than exhibiting a linear correlation between H3.3 nucleosome turnover and the transcriptional activity.

We then sorted the H3.3 nucleosomes by their corresponding turnover index (*T_48_*) from high to low. H3.3 nucleosomes that scored within the top 5% of the turnover index were defined as “high turnover” nucleosomes, and those scored within the bottom 5% of the turnover index were defined as “low turnover” nucleosomes. The “high turnover” H3.3 nucleosomes were relatively enriched at the promoters, 5′ UTRs and 3′ UTRs (Figure 2E), which is consistent with our observations in Figure 2C.

### Scoring the (H3.3–H4)_2_ tetramer splitting index

“Hybrid” mononucleosomes containing both newly synthesized Flag-H3.3 and old HA-H3.3 were purified and mapped according to experimental procedures described in Figure 1A. Then we developed a computational model to score the (H3.3–H4)_2_ tetramer splitting events and assigned an H3.3 nucleosome splitting index (*S*) for each H3.3 nucleosome (See the Materials and Methods for details).

Two challenges need to be addressed while developing the mathematic model for scoring the splitting events. Firstly, endogenous H3.3 histones exist in our system and they could form heterotetramers with both tagged versions, which cannot be monitored in our study. To solve this problem, we started with an adequate amount of cells, approximately 1.2×10^9^ cells (Figure S2B). The portion of detectable splitting events at any given genomic loci could be estimated to be [% Flag-tagged H3.3]×[% HA-tagged H3.3]×splitting rate, which should be approximately at the range of 10^−5^–10^−4^, because the tagged-H3.3 histones were at the level of approximately 5–10% total H3.3 histones and the global splitting rate was approximately 10% [22]. Therefore, for any given loci, we were able to capture approximately 10^4^∼10^5^ splitting events, which allowed us to study a representative population of the total splitting events and to obtain a relative measurement of the splitting rate.

**Figure 3:**
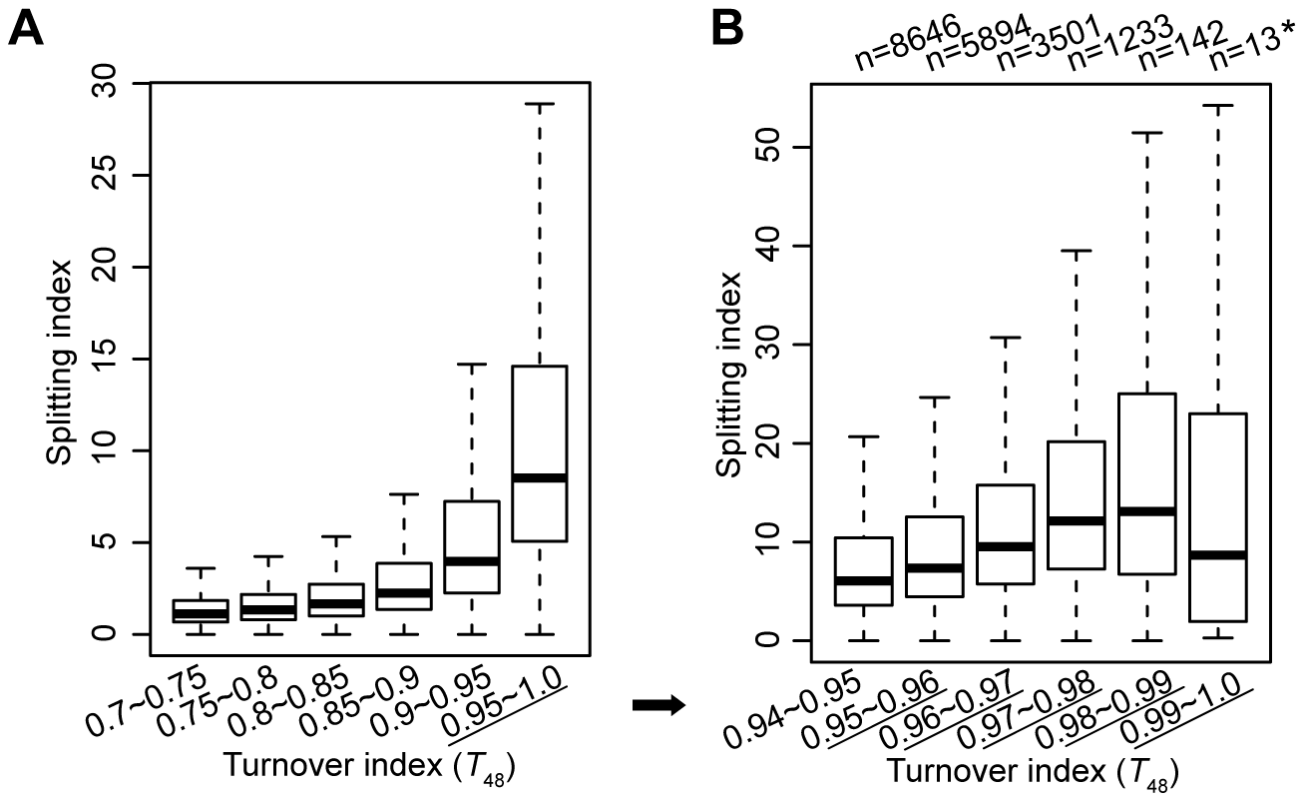
H3.3 nucleosomes with higher turnover index tend to associate with higher splitting index. (A) The distribution of splitting index for H3.3 nucleosomes within each specified turnover index range. (B) The distribution of splitting index for H3.3 nucleosomes within the highest turnover index ranges.

Secondly, at regions with the highest turnover rate, it is a concern that our approach may fail to capture the split nucleosomes. To clarify this concern, we categorized all H3.3 nucleosomes according to their turnover index (*T_48_*) range and compared their splitting index. H3.3 nucleosomes with higher turnover index clearly associated with higher splitting index (Figure 3A). To obtain an amplified view for H3.3 nucleosomes with the highest turnover rate, we further categorized these nucleosomes according to their turnover index (*T_48_*) range, and observed a similar trend, with the exception of the last group (*T_48_* range 0.99–1.0). But there were only 13 H3.3 nucleosomes defined in this group, and the results for this group may not be statistically meaningful. Taken together, H3.3 nucleosomes with the highest turnover index (*T_48_*) generally displayed the highest splitting index, suggesting that our approach could capture the splitting events at regions with the highest turnover.

### Moderate correlation between the H3.3 nucleosome turnover index and the splitting index

The above results suggested that H3.3 nucleosomes with higher turnover tend to associate with higher splitting events. To test the relationship between the H3.3 nucleosome turnover and splitting events further, we calculated the Pearson correlation between the turnover index (*T*) and splitting index (*S*) for all of the H3.3 nucleosomes and found a moderate positive correlation (*r*[*S*, *T*] = 0.3).

**Figure 4:**
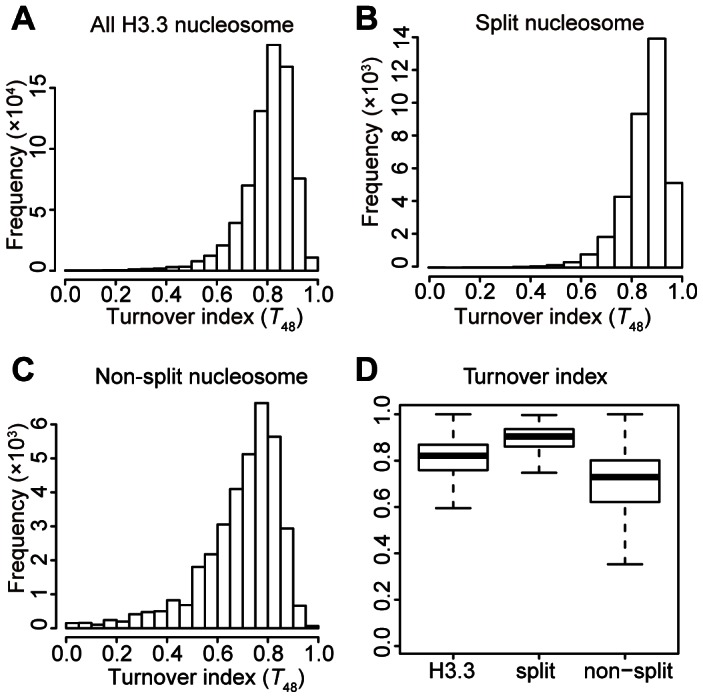
Moderate correlation between the H3.3 turnover index and splitting index. (A) Turnover index distribution profile for all H3.3 nucleosomes. (B) Turnover index distribution profile for the split H3.3 nucleosomes. (C) Turnover index distribution profile for the non-split H3.3 nucleosomes. (D) Box plot for the turnover index of all, split, and non-split H3.3 nucleosomes.

We then sorted all of the H3.3 nucleosomes by their corresponding splitting index (*S*), from high to low. The H3.3 nucleosomes scoring within the top 5% of the splitting index were defined as “split” nucleosomes, which possessed higher numbers of reads in the sequential ChIP than in the single-round ChIPs. The H3.3 nucleosomes scoring within the bottom 5% of the splitting index were defined as “non-split” nucleosomes, which possessed high numbers of reads in the single-round ChIPs but no reads in the sequential ChIP. Next, we calculated the frequency at a discrete turnover index range for the total H3.3 nucleosomes, the “split” nucleosomes and the “non-split” nucleosomes, respectively (Figure 4). The “non-split” H3.3 nucleosomes displayed a moderate lower turnover profile than the total H3.3 nucleosomes (Figure 4A, 4C and 4D), while the “split” H3.3 nucleosomes were slightly enriched at the high turnover range (Figure 4B and 4D). Nevertheless, the majority of non-split nucleosomes were within the high turnover range (Figure 4C–4D). These observations suggest that the H3.3 nucleosome splitting events are unlikely to be merely the consequence of the H3.3 nucleosome turnover.

### H3.3 nucleosome splitting events are even more enriched at active genes than the H3.3 nucleosomes themselves

**Figure 5:**
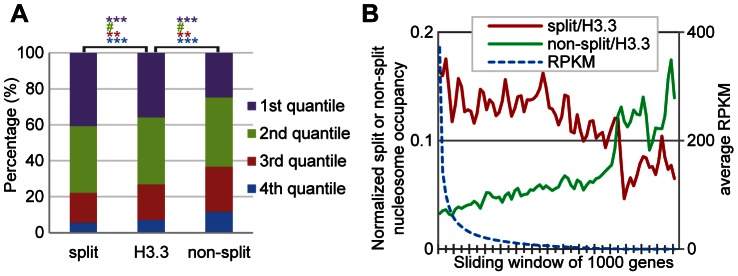
H3.3 nucleosome splitting events are better markers for active transcription than H3.3 nucleosome occupancy. (A) Split H3.3 nucleosomes were enriched in the top 25% expression level genes, as compared to the total H3.3 nucleosomes or non-split H3.3 nucleosomes. Non-split H3.3 nucleosomes were enriched in the bottom 25% expression level genes. P values were calculated with chi-square test. ***P<0.001, **P<0.01, #P>0.1. (B) After normalization against the H3.3 occupancy, the split but not the non-split H3.3 nucleosomes were enriched at active genes. H3.3 nucleosomes at the entire genes were analyzed together.

We next examined the relationship between the H3.3 nucleosome splitting events and transcriptional activity. For total H3.3 nucleosomes, split H3.3 nucleosomes (within the splitting index top 5%) and non-split H3.3 nucleosomes (within the splitting index bottom 5%) localized at genes, we individually examined their distribution profiles within different classes of genes that were categorized by their expression levels. Approximately 36% of total H3.3 nucleosomes and 25% of non-split H3.3 nucleosomes were localized at genes within the top 25% for expression levels. In contrast, 41% of the split H3.3 nucleosomes were located at these genes (Figure 5A), which is a significant difference with P value less than 0.0001 analyzed with Chi-square test. On the other hand, we found that 7% of total H3.3 nucleosomes and 5.5% of the split H3.3 nucleosomes were localized at genes within the bottom 25% for expression levels. However, 12% of non-split H3.3 nucleosomes were located at these genes (Figure 5A), which is also a significant enrichment with P value less than 0.0001. These data suggest that the split H3.3 nucleosomes were relatively enriched at active genes and non-split H3.3 nucleosomes were enriched at inactive genes.

For each gene, we scored its normalized split nucleosome occupancy (the number of split nucleosomes normalized against the number of H3.3 nucleosomes) and the non-split nucleosome occupancy (the number of non-split nucleosomes normalized against the number of H3.3 nucleosomes) and then plotted them against the RPKM. The split nucleosomes were enriched at the active genes, even after the normalization against the levels of H3.3 occupancy, while the non-split nucleosomes showed enrichment at the inactive genes (Figure 5B). Therefore, we conclude that the H3.3 nucleosome splitting events are better markers of active genes than H3.3 nucleosome occupancy.

### H3.3 nucleosome splitting events are enriched at cell-type specific enhancers

**Figure 6:**
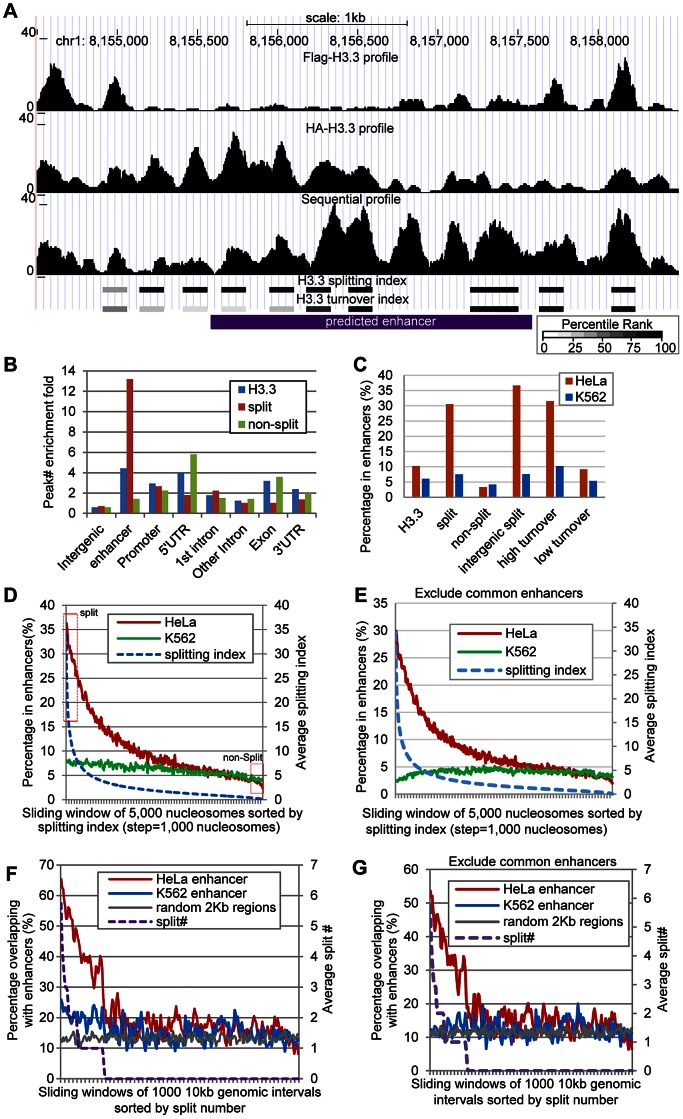
H3.3 nucleosome splitting events feature cell-type specific enhancers. (A) An example enhancer region enriched with split H3.3 nucleosomes. Profiles of single-round ChIPs, sequential ChIP, turnover index, splitting index are illustrated. Percentile ranking of turnover index and splitting index are shown in a grey scale. (B) Split H3.3 nucleosomes were specifically enriched at enhancers, whereas the non-split H3.3 nucleosomes were specifically depleted at enhancers. (C) Distribution of the H3.3 nucleosomes, split and non-split H3.3 nucleosomes, intergenic split H3.3 nucleosomes and high and low turnover H3.3 nucleosomes at the cell-type specific enhancers. (D) All H3.3 nucleosomes were sorted by their splitting index and grouped into 5000 nucleosome widows. These nucleosomes were then plotted against their overlap percentage with enhancers. The arbitrarily defined split and non-split nucleosomes with top or bottom 5% splitting index were boxed in red. (E) Similar to (D), but common enhancers were excluded. (F) The 10-kb genomic intervals sorted by their numbers of split nucleosomes were plotted against their overlap percentage with the cell-type specific enhancers. (G) Similar to (F), but common enhancers were excluded.

The H3.3 nucleosomes were reported to display cell-type specific enrichment at intergenic regions bound by multiple transcription factors, suggesting an enrichment of the H3.3 nucleosomes at the enhancers [9], which prompted us to interrogate the splitting events at the enhancers. Interestingly, it appears to be quite obvious that split H3.3 nucleosomes are enriched at a number of enhancers that we looked into (Figure 6A and Figure S3).

Genome-wide distribution of enhancers was previously determined in HeLa cells, based on distinct pattern of histone modifications, including enrichment of H3K4me1 and H3K27ac [27]. Because we used the same cells in this study to map the splitting events, we were able to examine the H3.3 nucleosome splitting events at these enhancers (each enhancer was arbitrarily defined as a 2 kb region). We first scored the relative enrichment for the H3.3 nucleosomes, the split H3.3 nucleosomes and the non-split H3.3 nucleosomes at various genomic features. Indeed, the H3.3 nucleosomes were enriched at the enhancers in HeLa cells, with a comparable fold-enrichment in the promoter regions and 5′ UTRs (Figure 6B). Strikingly, the split H3.3 nucleosomes were far more enriched at the enhancers than any of the other genomic features tested (Figure 6B). We found that 10% of the total H3.3 nucleosomes were located at the HeLa enhancers whereas 31% of the split H3.3 nucleosomes (within the splitting index top 5%) were located at the HeLa enhancers; in contrast, only 3% of the non-split H3.3 nucleosomes (within the splitting index bottom 5%) were located at the HeLa enhancers (Figure 6C). In addition, approximately 37% of all of the intergenic split H3.3 nucleosomes were specifically located at the HeLa enhancers, but such enrichment was not observed at the K562 cell-specific enhancers (Figure 6C). These data suggest that H3.3 nucleosome splittings are frequent events that feature cell-type specific enhancers.

In the above analysis, an arbitrary 5% cut off was employed. To obtain the continuity of this analysis, we sorted all H3.3 nucleosomes by their splitting index from high to low. Then we used a sliding window of 5000 H3.3 nucleosomes and analyzed the percentage of those nucleosomes that reside in the enhancers. The percentage of H3.3 nucleosomes reside in the HeLa enhancers declined continuously along with the reduction of their splitting index (Figure 6D). We noticed that H3.3 nucleosomes with high splitting index also displayed a minor enrichment at the K562 enhancers (Figure 6D). Interestingly, such enrichment was diminished when common enhancers between HeLa cells and K562 cells were excluded from the analysis (Figure 6E). This further supports that H3.3 splitting events are enriched at active enhancers in a cell-type specific manner.

To further investigate the relationship between the H3.3 nucleosome splitting events and enhancers, we divided the entire human genome into 10-kb intervals and sorted them by their split H3.3 nucleosome numbers. We then plotted the H3.3 nucleosome split number of these 10-kb intervals against their overlapping percentage with the enhancers. Those genomic intervals with higher numbers of split H3.3 nucleosomes clearly displayed a higher overlap with the HeLa enhancers but not the K562 enhancers (Figure 6F). Moreover, the overlapping percentage with the enhancers dropped to background level when the number of split H3.3 nucleosomes declined to zero. A minor overlap with K562 enhancers was observed for the genomic regions with high numbers of split H3.3 nucleosomes (Figure 6F), which was again diminished when the common enhancers between the two cell lines were excluded (Figure 6G).

We also sorted the 10-kb genomic intervals by their number of H3.3 nucleosomes, and then analyzed their overlap with enhancers. Those regions with high numbers of H3.3 nucleosomes were significantly enriched at the HeLa enhancers, but also at the K562 enhancers, regardless whether the common enhancers were excluded from the analysis or not (Figure S4). These data collectively suggest that the enrichment of H3.3 nucleosome splitting events, but not H3.3 occupancy, feature cell-type specific enhancers.

### Enrichment of H3.3 nucleosome splitting events is not the mere consequence of H3.3 nucleosome turnover

Considering the high turnover H3.3 nucleosomes (within the turnover index top 5%) were also enriched at enhancers (Figure 6C), and H3.3 nucleosome splitting index displayed modest positive correlation with turnover index (*T_48_*) (Figure 3 and Figure 4), it is necessary to examine the correlation between the splitting events and turnover at the enhancers.

**Figure 7:**
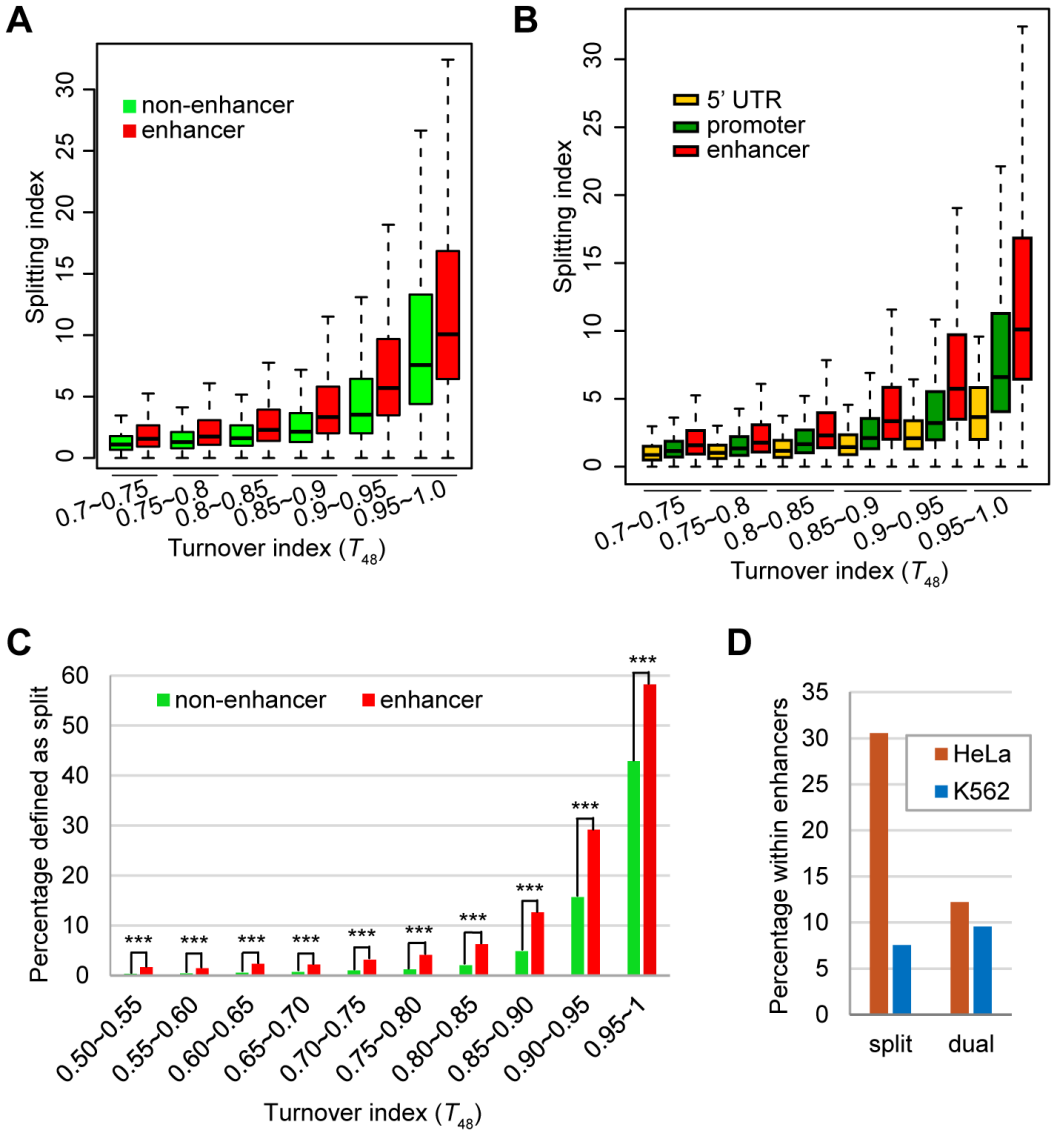
The enhancer H3.3 nucleosomes display higher splitting index than the non-enhancer H3.3 nucleosomes. (A) Box plot of the splitting index of enhancer or non-enhancer H3.3 nucleosomes within the same turnover ranges. (B) Box plot of the splitting index of H3.3 nucleosomes at the enhancers, promoters, 5-UTRs within the same turnover ranges. (C) Percentage of split nucleosomes for enhancer H3.3 or non-enhancer H3.3 at various turnover ranges. *** indicates the significant difference with P value<0.0001. (D) Dual-tagged H3.3 nucleosomes derived from the co-expression experiment did not show enrichment at cell-type specific enhancers.

We first categorized H3.3 nucleosomes into two groups, the enhancer group (all H3.3 nucleosomes reside in enhancers) and the non-enhancer group (all H3.3 nucleosomes do not reside in enhancers), and then we compared the splitting index of these two groups within the same turnover range. Interestingly, enhancer H3.3 nucleosomes displayed higher split index than non-enhancers H3.3 nucleosomes with similar turnover index (Figure 7A).

We then performed similar comparison for three groups of H3.3 nucleosomes located at the enhancers, promoters and 5′-UTRs, because these regions displayed comparable enrichment of H3.3 nucleosomes (Figure 6B). H3.3 nucleosomes at the enhancers were clearly associated with higher splitting index than H3.3 nucleosomes at the promoters or 5′-UTRs with similar turnover index (Figure 7B).

Furthermore, for H3.3 nucleosomes at comparable turnover ranges, significantly higher percentage of enhancer H3.3 nucleosomes were called as “split” nucleosomes than the non-enhancer H3.3 nucleosomes (Figure 7C). These results support the notion that splitting events are not merely the consequence of nucleosome turnover and there might be active splitting mechanism(s) at the enhancers. We also noticed that the percentage of “split” nucleosomes displayed a difference greater than two-fold between the enhancer group and the non-enhancer group at most turnover range (Figure 7C), except for H3.3 nucleosomes with the highest turnover (*T_48_* between 0.95 and 1), but the difference remained to be statistically significant (Figure 7C). These results are consistent with our observation that high turnover (Figure 3 and Figure 7) and enhancer enrichment (Figure 6 and Figure 7) are both associated with “split” H3.3 nucleosomes.

Finally, to exclude any potential bias of our sequential ChIP experiments, we co-expressed both tagged H3.3 nucleosomes at the same time and then performed single-round and sequential ChIP-Seq experiments (Figure S2D). As expected, profiles of the single-round and sequential ChIP-Seq results were highly similar (Figure S5) and no specific enrichment of dual-tagged H3.3 nucleosomes was observed at the HeLa enhancers (Figure 7D).

## Discussion

Active genes have higher H3.3 nucleosomes occupancy [5], [6], [9] and higher nucleosome turnover [26]. In this report, we found that active genes are also associated with higher H3.3 nucleosome turnover and higher H3.3 nucleosome splitting events. But what are the relationships among all these events? Do they simply reflect one event at the active genes or they might have different roles? Recently, the yeast nucleosomes, which consist the “H3.3-like” H3 histones [3] were shown to display some level of splitting events [28], similar to the human H3.3 nucleosomes [22]. In yeast, active genes also tended to have higher nucleosome splitting signals [28], similar to our observation (Figure 5). Actively transcribing genes have a higher nucleosome turnover and a higher nucleosome splitting; therefore it appears to be logical to think that these events might be directly correlated with each other. However, by comparing these parameters at the genome-wide level, we found that, although these events are indeed correlated with active transcription, there is only a moderate correlation between the H3.3 nucleosome turnover and the H3.3 nucleosome splitting events (Figure 3 and Figure 4). Nucleosomes with the same turnover index exhibited different splitting indexes at enhancer regions and non-enhancer regions (Figure 7), which also suggests these events are not directly correlated. We believe that this is because of the fact that neither of these events is linearly correlated with the transcriptional activity (Figure 3D and Figure 5B).

The surprising observation that H3.3 nucleosomes with high splitting index were remarkably enriched at cell-type specific enhancers (Figure 6 and Figure 7) may suggest a role for nucleosome splitting in regulating cell-type specific transcription, especially when these splitting events are clearly not the mere consequence of H3.3 nucleosome turnover (Figure 7). We propose the existence of active mechanism(s) at cell-type specific enhancers, which may regulate lineage-specific transcription.

This study represents a first attempt to unveil the role of H3.3 nucleosome splitting events. The unexpected observation of the enrichment of these events at enhancers is highly interesting. However, there are more questions than answers at this stage regarding the functional significance and molecular mechanism of this observation. Function-wise, it would be interesting to ask whether this event is related to the transmission of epigenetic modifications, as previously proposed [25] or whether this event maybe relevant to cell fate determination. One interesting experiment is to transplant the current detection system into the stem cell systems, and to ask whether splitting events may localize differently in cells at the self-renewal stage and differentiated stage. It is also highly interesting to understand the molecular mechanism of the splitting events and even to manipulate the splitting events. We speculate that the H3.3 deposition chaperones and the chromatin remodelers may participate in the splitting events. However, it is highly challenging to draw a firm conclusion without a strategy that uncouples the splitting events and the H3.3 nucleosome deposition pathway.

## Materials and Methods

### Cell lines

Stable HeLa cells expressing Flag-H3.3 histones under the control of a tetracycline-inducible promoter were established in our previous work [22]. These cells were stably engineered with the pIND system (Invitrogen) to express the HA-H3.3 histones under the control of a Ponasterone A-inducible promoter.

### ChIP-Seq

Mononucleosomes were prepared, according to the literature [29], using 1.2×10^9^ cells that were sequentially induced (Figure 1B). Affinity purified mononucleosomes were eluted with Flag or HA peptides according to our previous reports [21], [22]. DNA samples were extracted from affinity-purified mononucleosomes by phenol extraction and ethanol precipitation. Solexa sequencing libraries were constructed with the NEBNext DNA Sample Prep Master Mix Set 1 Kit following the manufacturer's instructions, and then subjected to single-end sequencing on Illumina Genome Analyzer II.

### Identification of H3.3 nucleosomes

The sequencing reads of Flag-H3.3 (new) and HA-H3.3 (old) were pooled and shifted to the center of fragment size of 150 bp. The middle half size (i.e. 75 bp) of each read was kept for the following analysis. The bigwig profile was generated with the NPS program [30]. A 150 bp-window was used to scan the hg19 genome with a 10 bp step. The window was defined as a nucleosome if the reads profile met the following criterions: the middle point of the window was the highest or the 2^nd^ highest and greater than 9; the middle point was greater than the 30% percentile of the window. For nucleosomes with the distance of 10 bp, the more symmetric one was kept. “Adjacent” nucleosomes called by the program with more than 50% overlap were considered as fuzzy nucleosomes and excluded from further analysis.

### RNA-Seq

The RNA-Seq experiment was performed according to a previous publication [31]. The sequencing reads were mapped to human genome hg19 using Tophat [32], and the RPKM values were quantified using Cufflinks [33].

### Definitions and formulae for calculating the H3.3 nucleosome turnover index and splitting index

To measure turnover index at relatively late time points required for the splitting assay, adequate amount of starting cells (approximately 1×10^8^ cells) were used for ChIP-Seq experiments illustrated in Figure 2A. The turnover index was calculated by comparing HA-H3.3 profiles at time point *t* (*H_t_*) and time point 0 h (*H_0_*). For each H3.3 nucleosome, the signal ratio between the two time points (*H_t_/H_0_*) was defined as *R_48_* or *R_24_*. For each time point *t*, the signal ratio at H3.3 nucleosome with the lowest turnover in the genome was defined as *R_t,max_*; likewise, the signal ratio at H3.3 nucleosome with the highest turnover in the genome was defined as *R_t,min_*. To normalize the final turnover index (*T_t_*) to the range of 0 to 1, the turnover index for each H3.3 nucleosome was calculated as:
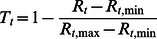
(1)For a given genomic location, the amount of dual-tagged “hybrid” nucleosomes (*D*) sequentially purified according to Figure 1B can be determined by the amounts of Flag-H3.3 nucleosome (*F*), HA-H3.3 (*H*), total available H3.3 nucleosomes (*N*) and the splitting index (*S*) using the following equation.
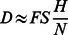
(2)The variables *F*, *H* and *D* were determined according to the ChIP-seq results from the Flag single-round, HA single-round and sequential ChIP-seq experiments. The variable *N* cannot be directly determined, but the amount of total available H3.3 nucleosomes should be proportional to the HA-H3.3 nucleosomes after a long period (48 h) of induction (*H_0_*), which has been determined in 0 h time point according to experiments described in Figure 2A. Therefore, 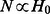, and:
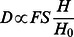
(3)and:
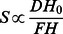
(4)


### Accession number

ChIP-Seq data have been deposited in the NCBI Sequence Read Archive under accession No. SRA043915. (http://www.ncbi.nlm.nih.gov/Traces/sra/sra.cgi)

## Supporting Information

Figure S1 Kinetics of the turn-on/turn-off rate of Flag-, HA-tagged H3.3 histones. (A). Experimental scheme for Figure S1E. (B–D). RT-PCR experiments showing the rapid turn-on of Flag- and HA-tagged H3.3 and the rapid turn-off of HA-tagged H3.3 at the mRNA level. mRNA levels were normalized against the mRNA levels of GAPDH. (E). Relative slow turnover of HA-H3.3 at the global level provides adequate amounts of HA-H3.3 histones at the time points we used for sampling the turnover events. Whole cell lysates from equal number of starting cells were used for the western blot analysis. Treatment of protein synthesis inhibitor Cycloheximide (CHX) along with Ponasterone A withdraw did not change the level of HA-H3.3 proteins (compare lane 5 and 6), indicating there was little residual synthesis of new HA-H3.3 proteins.
(PDF)

Figure S2 Quality controls of the experimental system. (A) DNA samples extracted from mononucleosomes on a 2% agarose gel. (B) Scale of the sequential-ChIP experiment. (C) Original gels showing that sequencing libraries were size fractionated prior to sequencing. Adapters with 92 bp were ligated to the DNA samples. Therefore we fractionated 200–300 bp library DNA samples to ensure DNA samples were originated from mononucleosomes. (D) Basic stats of the ChIP-Seq results.
(PDF)

Figure S3 Four regional examples. (A–C) Three examples of enhancer regions with high split H3.3 nucleosomes. HeLa-specific enhancers were included in panel A and B, while a HeLa/K562 common enhancer was included in panel C. (D) One example of genomic region with low splitting events. Region in green indicated a K562-specific enhancer. For A–D, H3K27Ac profiles of HeLa (light blue, data from Bing Ren) and K562 (dark blue, data from ENCODE project) were showed.
(PDF)

Figure S4 H3.3 nucleosome splitting events feature cell-type specific enhancers. (A) All 10-kb genomic intervals were sorted by their H3.3 nucleosome numbers and grouped into 1000 genomic interval windows. These windows were then plotted against their overlap percentage with enhancers. Regions with high H3.3 numbers were enriched at both HeLa and K562 cell enhancers. (B) Similar to (A), but excluded the common enhancers between these two cell lines.
(PDF)

Figure S5 Distribution profiles of co-expressed Flag-H3.3. (A), HA-H3.3 (B) and dual-tagged H3.3 (C) nucleosomes.
(PDF)
