# Supplementary figures and images for: H3.3-H4 Tetramer Splitting Events Feature Cell-Type Specific Enhancers
Source: PLoS Genet. 2013 Jun 6;9(6):e1003558. doi: 10.1371/journal.pgen.1003558 (PMC3675017; doi:10.1371/journal.pgen.1003558)

**A**

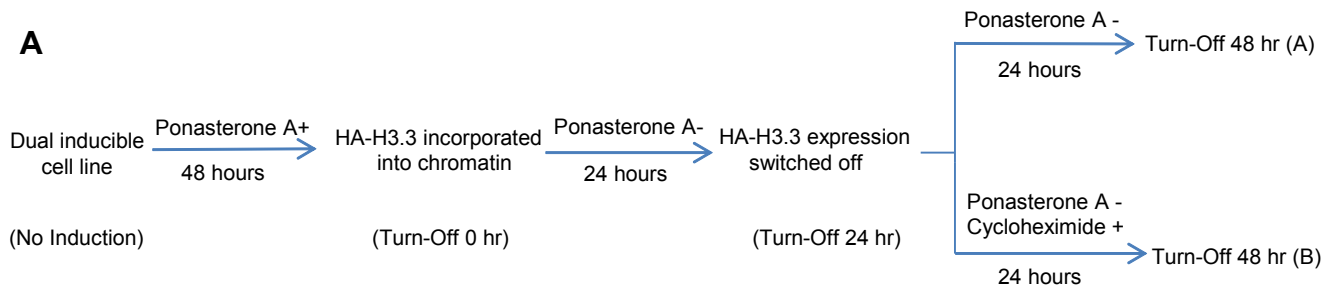

**B**

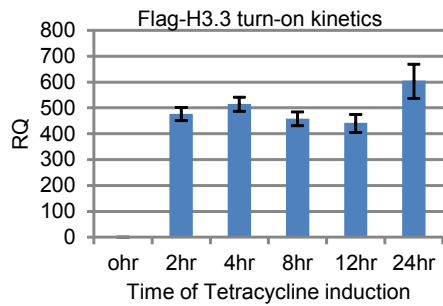

**C**

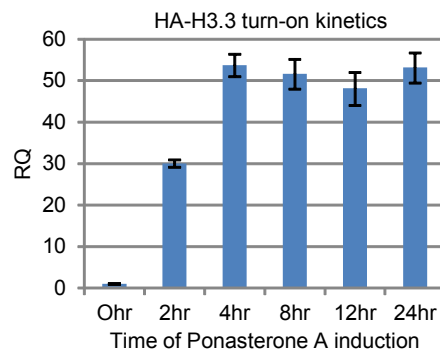

**D**

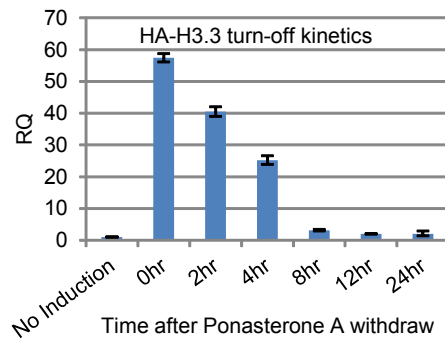

**E**

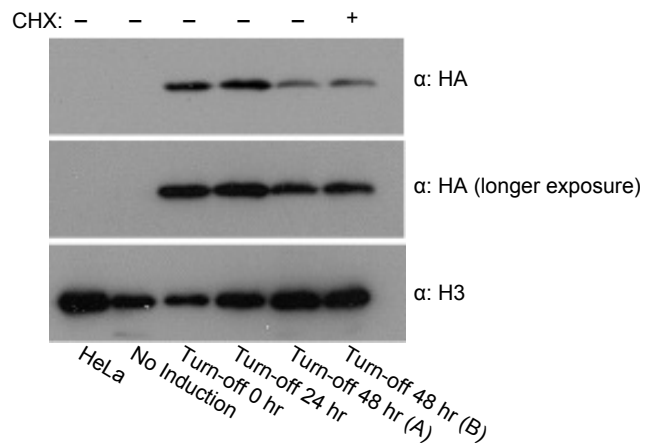

Supplement: Figure S1 — Kinetics of the turn-on/turn-off rate of Flag-, HA-tagged H3.3 histones. (A). Experimental scheme for Figure S1E. (B–D). RT-PCR experiments showing the rapid turn-on of Flag- and HA-tagged H3.3 and the rapid turn-off of HA-tagged H3.3 at the mRNA level. mRNA levels were normalized against the mRNA levels of GAPDH. (E). Relative slow turnover of HA-H3.3 at the global level provides adequate amounts of HA-H3.3 histones at the time points we used for sampling the turnover events. Whole cell lysates from equal number of starting cells were used for the western blot analysis. Treatment of protein synthesis inhibitor Cycloheximide (CHX) along with Ponasterone A withdraw did not change the level of HA-H3.3 proteins (compare lane 5 and 6), indicating there was little residual synthesis of new HA-H3.3 proteins. (PDF) [file pgen.1003558.s001.pdf]

Huang\_Figure S3

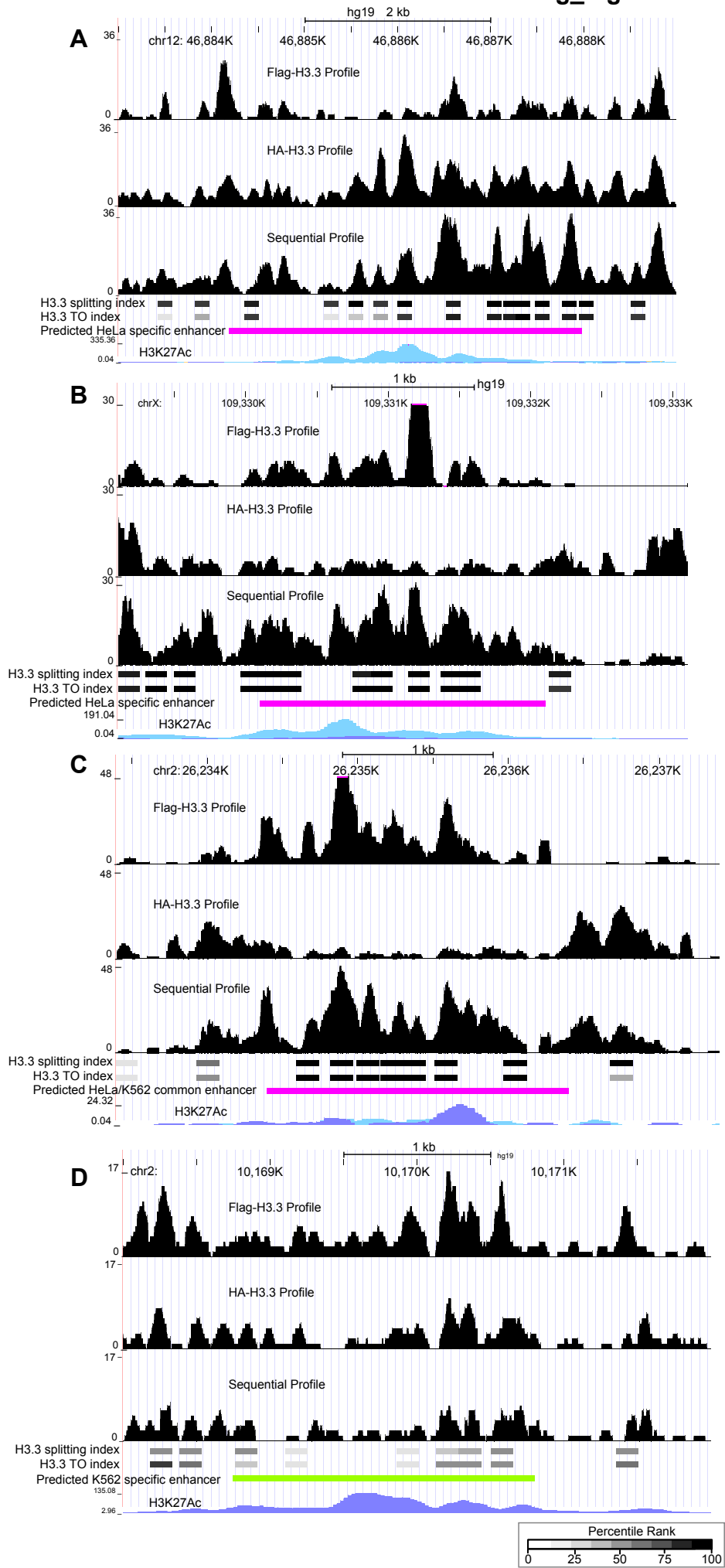

Supplement: Figure S3 — Four regional examples. (A–C) Three examples of enhancer regions with high split H3.3 nucleosomes. HeLa-specific enhancers were included in panel A and B, while a HeLa/K562 common enhancer was included in panel C. (D) One example of genomic region with low splitting events. Region in green indicated a K562-specific enhancer. For A–D, H3K27Ac profiles of HeLa (light blue, data from Bing Ren) and K562 (dark blue, data from ENCODE project) were showed. (PDF) [file pgen.1003558.s003.pdf]

Huang\_Figure S4

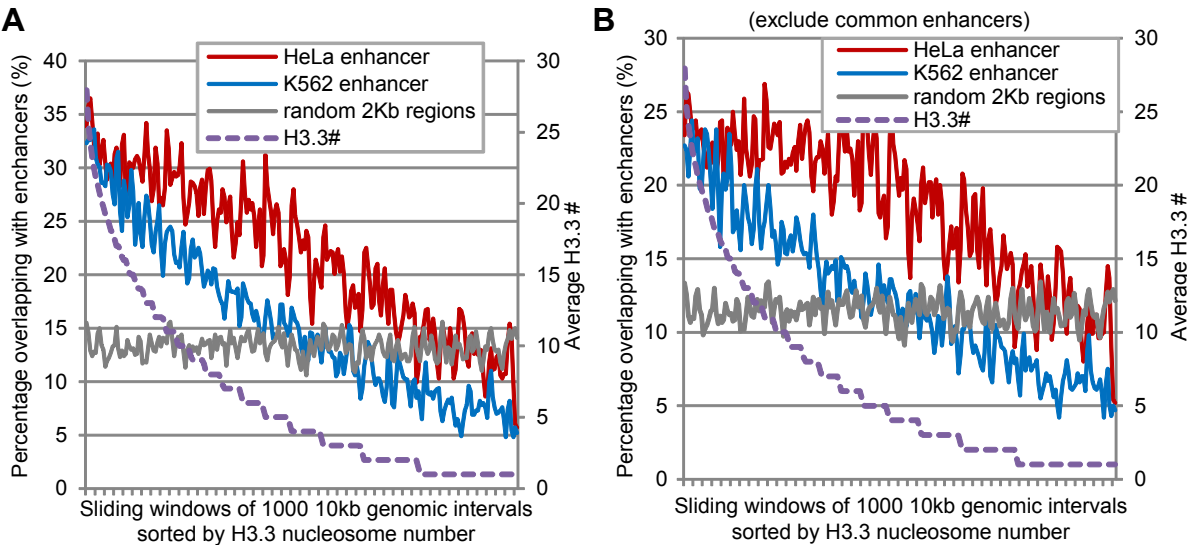

Supplement: Figure S4 — H3.3 nucleosome splitting events feature cell-type specific enhancers. (A) All 10-kb genomic intervals were sorted by their H3.3 nucleosome numbers and grouped into 1000 genomic interval windows. These windows were then plotted against their overlap percentage with enhancers. Regions with high H3.3 numbers were enriched at both HeLa and K562 cell enhancers. (B) Similar to (A), but excluded the common enhancers between these two cell lines. (PDF) [file pgen.1003558.s004.pdf]

Huang\_Figure S5

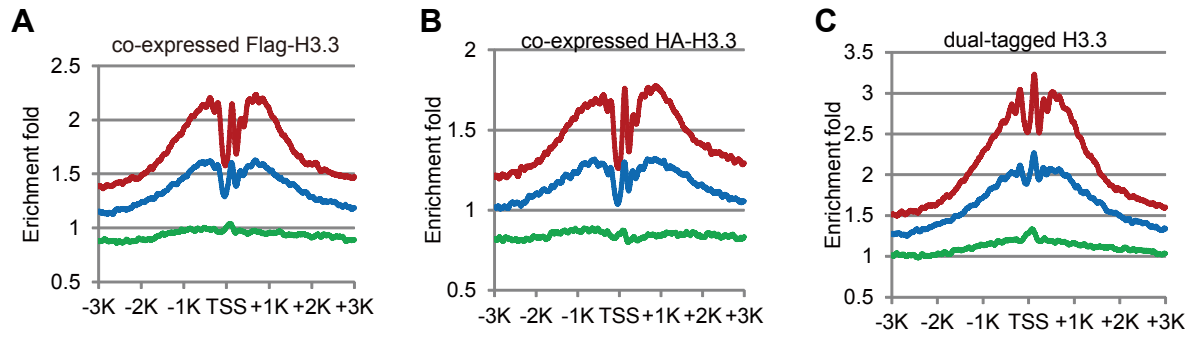

Supplement: Figure S5 — Distribution profiles of co-expressed Flag-H3.3. (A), HA-H3.3 (B) and dual-tagged H3.3 (C) nucleosomes. (PDF) [file pgen.1003558.s005.pdf]
