# Supplementary material for: H3.3-H4 Tetramer Splitting Events Feature Cell-Type Specific Enhancers
Source: PLoS Genet. 2013 Jun 6;9(6):e1003558. doi: 10.1371/journal.pgen.1003558 (PMC3675017; doi:10.1371/journal.pgen.1003558)
Supplement: Figure S2 — Quality controls of the experimental system. (A) DNA samples extracted from mononucleosomes on a 2% agarose gel. (B) Scale of the sequential-ChIP experiment. (C) Original gels showing that sequencing libraries were size fractionated prior to sequencing. Adapters with 92 bp were ligated to the DNA samples. Therefore we fractionated 200–300 bp library DNA samples to ensure DNA samples were originated from mononucleosomes. (D) Basic stats of the ChIP-Seq results. (PDF) [file pgen.1003558.s002.pdf]

Huang_Figure S2

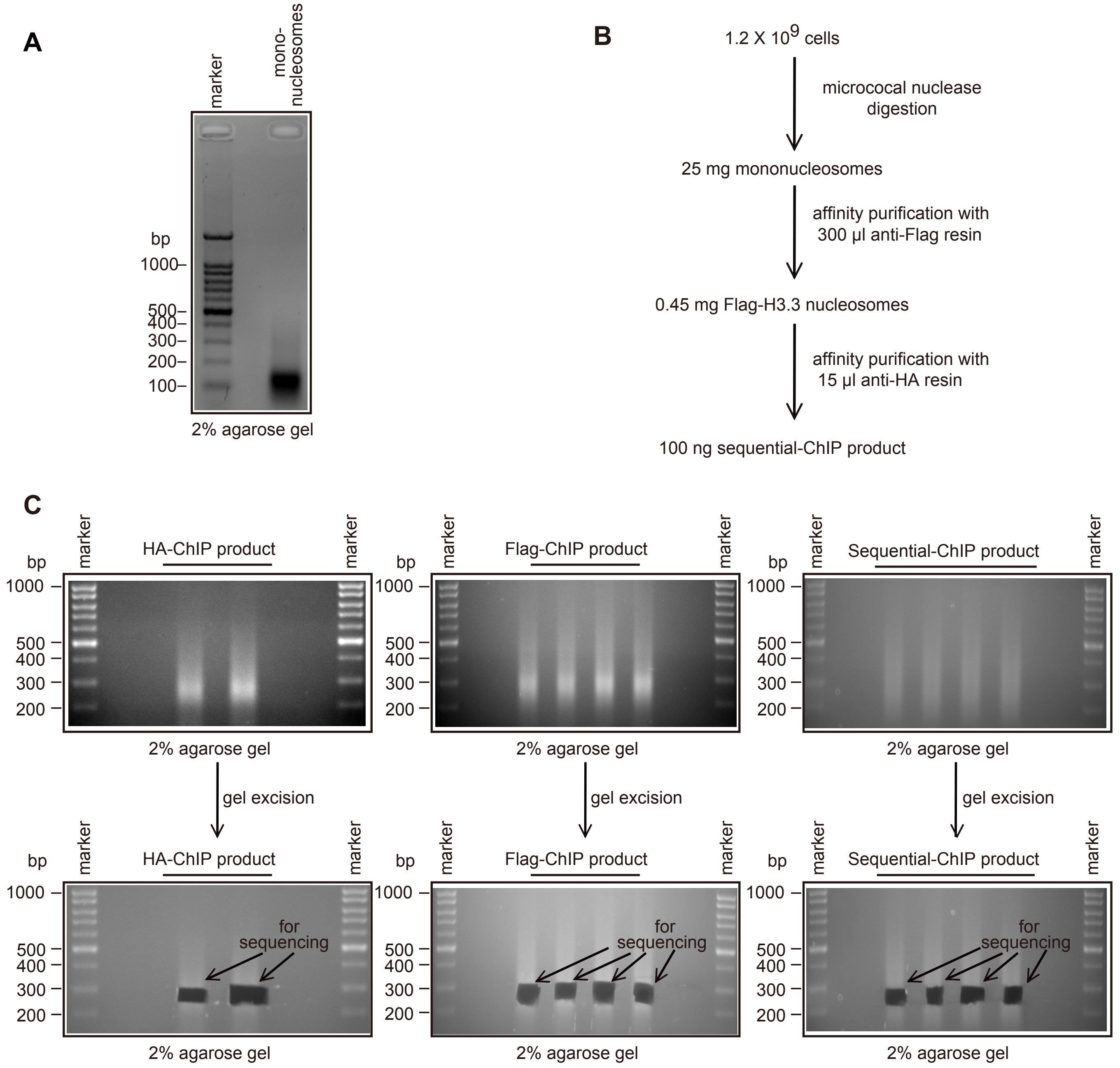

D

	sample	reads#	uniquely mapped	%
Fig. 1B	HeLa mononucleosome	34,515,426	27,911,860	80.87
	Flag-H3.3 single	56,049,533	44,215,584	78.89
	HA-H3.3 single	66,103,614	49,687,080	75.17
	Sequential-H3.3	69,106,522	45432288	65.74
Fig. 2A	HA-H3.3 0 h	126,204,390	87,015,169	68.95
	HA-H3.3 24 h	133,385,975	86,623,446	64.94
	HA-H3.3 48 h	168,648,856	114,834,109	68.09
Fig. 7D	co-expressed Flag-H3.3	140,021,966	96,200,122	68.70
	co-expressed HA-H3.3	142,889,495	87,124,158	60.97
	co-expressed Double-H3.3	143,515,150	57,284,102	39.92
